# The Complete Mitochondrial Genome of *Delia antiqua* and Its Implications in Dipteran Phylogenetics

**DOI:** 10.1371/journal.pone.0139736

**Published:** 2015-10-01

**Authors:** Nai-Xin Zhang, Guo Yu, Ting-Jing Li, Qi-Yi He, Yong Zhou, Feng-Ling Si, Shuang Ren, Bin Chen

**Affiliations:** Institute of Entomology and Molecular Biology, College of Life Sciences, Chongqing Normal University, Chongqing, 401331, China; Sichuan University, CHINA

## Abstract

*Delia antiqua* is a major underground agricultural pest widely distributed in Asia, Europe and North America. In this study, we sequenced and annotated the complete mitochondrial genome of this species, which is the first report of complete mitochondrial genome in the family Anthomyiidae. This genome is a double-stranded circular molecule with a length of 16,141 bp and an A+T content of 78.5%. It contains 37 genes (13 protein-coding genes, 22 tRNAs and 2 rRNAs) and a non-coding A+T rich region or control region. The mitochondrial genome of *Delia antiqua* presents a clear bias in nucleotide composition with a positive AT-skew and a negative GC-skew. All of the 13 protein-coding genes use ATN as an initiation codon except for the *COI* gene that starts with ATCA. Most protein-coding genes have complete termination codons but *COII* and *ND5* that have the incomplete termination codon T. This bias is reflected in both codon usage and amino acid composition. The protein-coding genes in the *D*. *antiqua* mitochondrial genome prefer to use the codon UUA (Leu). All of the tRNAs have the typical clover-leaf structure, except for *tRNA* ^*Ser(AGN)*^ that does not contain the dihydrouridine (DHU) arm like in many other insects. There are 7 mismatches with U-U in the tRNAs. The location and structure of the two rRNAs are conservative and stable when compared with other insects. The control region between *12S rRNA* and *tRNA* ^*Ile*^ has the highest A+T content of 93.7% in the *D*. *antiqua* mitochondrial genome. The control region includes three kinds of special regions, two highly conserved poly-T stretches, a (TA)_n_ stretch and several G(A)_n_T structures considered important elements related to replication and transcription. The nucleotide sequences of 13 protein-coding genes are used to construct the phylogenetics of 26 representative Dipteran species. Both maximum likelihood and Bayesian inference analyses suggest a closer relationship of *D*. *antiqua* in Anthomyiidae with Calliphoridae, Calliphoridae is a paraphyly, and both Oestroidea and Muscoidea are polyphyletic.

## Introduction

The mitochondrion is an important organelle in eukaryotic cells. It is connected with energy metabolism, apoptosis, aging, and disease and is a location for oxidative phosphorylation [1]. The mitochondrion is known as the cell's "powerhouse" or "power station" because it provides energy for cells through oxidative phosphorylation. The growth and proliferation of mitochondria are controlled by both the nuclear genome and its own genome, so it is considered a semi-autonomous organelle [2].

The mitochondrial genome is a covalently closed circular double-stranded molecule with a small molecular weight. It has a high copy number, does not contain introns, has a compact gene arrangement, and is lack of recombination [3]. There are significant differences in the size of the mitochondrial genome among different organisms. The insect mitochondrial genome is 13–19 kb in length and is composed of an encoding region containing 37 genes (13 protein coding genes, 22 tRNA genes and 2 rRNA genes) and a non-coding A+T rich region. The non-coding A+T rich region, also called as the control region (CR), is considered to control the replication and transcription of the mitochondrial genome [4]. The length variation among insect mitochondrial genomes is mainly determined by variation in the A+T rich region, which varies from 70 to 13 kb in length [5].

The mitochondrial genome is widely reported for its difference from the nuclear genome in its nucleotide composition, codon usage, gene sequencing and tRNA secondary structure [6–8]. Mitochondrial genomes are widely used in phylogenetics as well as in the study of the comparative and evolutionary genomics of insects. Mitochondrial genomes are also ideal molecular markers in population genetics and molecular evolution. All of these are due to mitochondria having a matrilineal inheritance, lack of extensive recombination, a conservative gene structure and composition, a low mutation rate and a faster evolution than nuclear genomes [9–10]. In recent years, partial coding genes of the mitochondrial genome, such as *COI*, *COII*, have become widely used in molecular phylogenetic analysis. The genome order has also been used as genetic markers to solve the phylogenetic relationships among distantly related taxa [11].

Insects exhibit the most extensive range of taxa on the planet, and insects have also been the subject of more research than other species. To date, there are more than 480 insect mitochondrial genome sequences published, among which there are 77 complete or nearly complete sequences from Diptera [12], accounting for 16% of the total sequences. These dipteran mitochondrial genome sequences provide an important database reference and are the basis for new molecular phylogenetic analyses of insects.

The onion maggot *Delia antiqua*, belonging to the family Anthomyiidae in the superfamily Muscoidea, is a major underground agricultural pest with wide distributed in Asia, Europe and North America. Its larvae damage bulb onions, garlic, chives, shallots, leeks and the bulbs of tulips, and reside in rotting liliaceous vegetables [13]. It naturally enters diapause in the pupal stage in summer or winter seasons just after the head evagination completed, and can serve a good model for insect diapause study [14]. To date, the mitochondrial genome sequence of this species has not been available. The Muscoidea was considered to be a paraphyly and the superfamily Oestroidea was nested within the Muscoidea. The phylogenetic relation of the two superfamilies and the location of Anthomyiidae are still not resolved [15–17].

In this study, we report the complete mitochondrial genome sequence, and investigate the organization, composition, codon usage and RNA secondary structure of the *Delia antiqua* and kown dipteran mitochondrial genomes. Importantly, this is the first report and description of complete mitochondrial genome of the family Anthomyiidae. We constructed the phylogenetic relationship of 26 representative species of known dipteran mtgenomes, and provide new insight in the phylogenetics of the two superfamilies. We found that Anthomyiidae was claded in Calliphoridae in the Oestroidea.

## Materials and Methods

### Sampling and DNA Extraction

*Delia antiqua* colony was reared in the Institute of Entomology and Molecular Biology, Chongqing Normal University, China at 20 ± 0.2°C under 50–70% relative humidity with a 16L:8D photocycle as previously described [13]. The mitochondrial genomic DNA was extracted from the third instar of larvae with the TIANamp Genomic DNA Kit (TianGen, China).

### PCR Amplification and Sequencing

The mitochondrial genome of *D*. *antiqua* was amplified by overlapping short PCR fragments (<1.2kb) with the extracted genomics DNA. All 26 fragments were amplified using the universal primers for Diptera designed by Zhang et al [18]. All short PCRs were carried out using Takara rTaq DNA polymerase (Takara, China) under the following cycling conditions: denaturation at 94°C for 5 min, followed by 35 cycles of denaturation at 94°C for 40 s, annealing at 48–55°C for 45 s, and elongation at 72°C for 1 min. The final elongation step was continued for 10 min at 72°C. These PCR products were analyzed by 1.0% agarose gel electrophoresis. All amplified products were sequenced directly except for the control region, which was sequenced after cloning into pMD-19T Vector. All fragments were sequenced in both directions.

### Sequence Assembly, Annotations and Analysis

Sequences obtained were assembled using DANMAN (http://www.lynnon.com/). Protein-coding genes were aligned by Clustal X [19], then identified and translated to amino acids through MEGA version 4.0 [20]. rRNA genes were identified by sequence comparison with other dipteran insect [21]. Almost all tRNAs were recognized by tRNAscan-SE Search Server v.1.21 online [22] and the tRNAs that could not be found by tRNAscan-SE were confirmed by sequence comparison with other dipteran insects. The control region was examined for repeats and special structures with the aid of the Tandem Repeats Finder (http://www.bioinfo.rpi.edu/applications/Mfold) [23]. The nucleotide composition was calculated by the DNA Star (http://www.dnastar.com/, [24]). The relative synonymous codon usage was calculated by MEGA version 4.0 [20]. Strand asymmetry was evaluated by AT Skew and GC Skew using the formulae: AT skew = [A% − T%] / [A% + T%] and GC skew = [G% − C%] / [G% + C%] [22].

### Phylogenetic Analysis

Phylogenetic analysis was carried out based on 26 complete mitochondrial genome sequences from the known 75 dipteran sequences. *Bombyx mandarina* was selected as the out-group (S1 Table). Phylogenetic trees were built based on the 13 protein-coding genes. First, the alignment of amino acids for every protein-coding gene was carried out using Clustal X [19]. Then, we concatenated the alignment results of individual genes. Model selection was done with Modeltest 3.7 [25] and MrModeltest 2.3 [26] for ML analysis and Bayesian inference, respectively. The results showed that the GTR+I+G model was the most ideal for analysis using nucleotide alignments. The GTR+I+G model was used with MrBayes Version 3.1.1 [27] and a PHYML online web server [28]. The alignments were used to carry out a maximum likelihood (ML) and Bayesian analysis (BI), using PHYML [28] and MrBayes [27]. In Bayesian analysis, the average standard deviation of split frequencies was below 0.01, and about 1,000,000 generations were conducted for the matrix, and each set was sampled every 200 generations with a burn of 25%. Finally, we removed the aging trees and exported the optimal tree.

## Results and Discussion

### Genome Organization

**Fig 1:**
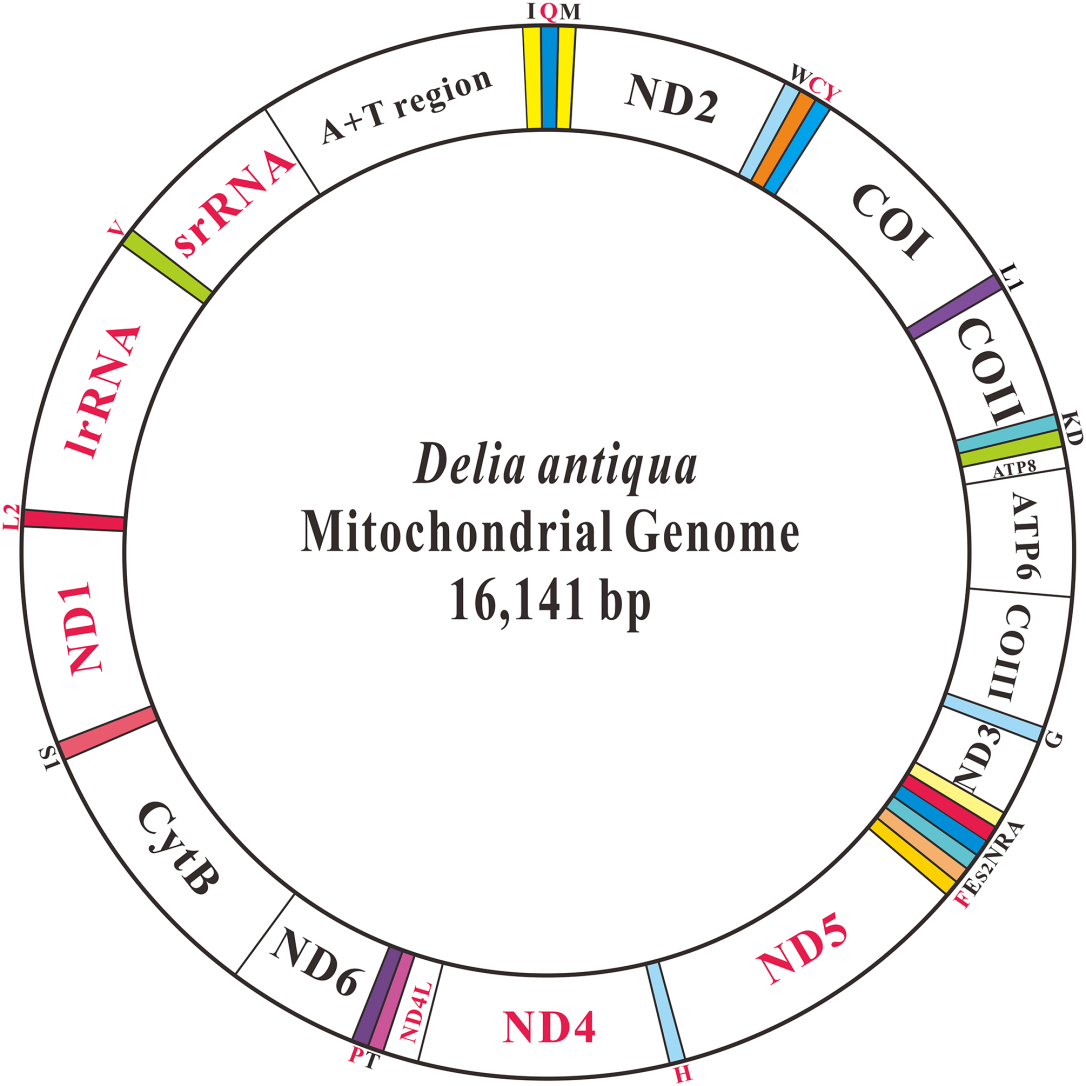
The structure of the mitochondrial genome of *Delia antiqua*. The color-filled blocks indicate tRNAs, while the un-filled white blocks denote protein-coding genes, rRNA and control regions. The protein-coding genes, rRNA and control regions with black name are located on majority strand, whereas those with red names are on minority strand.

The complete mitochondrial genome of *D*. *antiqua* is a double stranded circular molecule with a length of 16,141 bp (Fig 1, GenBank accession number KT026595). The genome is medium-sized in compared with other Diptera mitochondrial genomes that range from 14,503 bp (*Rhopalomyia pomum*) to 19,517 bp (*Drosophila melanogaster*) in length. It includes 37 genes (13 protein coding genes, 22 tRNAs and 2 rRNAs) and a non-coding region (A+T rich region, also called as the control region) (Table 1). There are 23 genes located on the J-strand (9 protein coding genes and 14 tRNAs) with the other 14 genes on the N-strand (4 protein coding genes, 8 tRNAs and 2 rRNAs). Fourteen intergenic spacers were found to have a total length of 127 bp, ranging in size from 2–26 bp and with the longest intergenic spacer located between *tRNA* ^*Arg*^ and *tRNA* ^*Asn*^. On the other hand, there were 12 gene overlaps in the mitochondrial genome of *D*. *antiqua* and they involve in a total of 43 bp; the longest overlap was 8 bp and appears between *tRNA* ^*Trp*^ and *tRNA* ^*Cys*^.

**Table 1:** Organization of the *Delia antiqua* mitochondrial genome.

Gene	Direction	Location	Size (bp)	Anticodon	Codon		Intergenic nucleotides^a^
					Start	Stop	
*tRNA* ^*Ile*^	F	1–66	66	31-33GAT			
*tRNA* ^*Gln*^	R	64–132	69	100-102TTG			-3
*tRNA* ^*Met*^	F	136–204	69	166-168CAT			3
*ND2*	F	205–1221	1017		ATT	TAA	0
*tRNA* ^*Trp*^	F	1220–1287	68	1250-1252TCA			-2
*tRNA* ^*Cys*^	R	1280–1342	63	1311-1313GCA			-8
*tRNA* ^*Tyr*^	R	1353–1418	66	1385-1387GTA			10
*COI*	F	1416–2967	1552		ATCA	TAA	-3
*tRNA* ^*Leu(UUR)*^	F	2963–3028	66	2992-2994TAA			-5
*COII*	F	3037–3724	688		ATG	T	8
*tRNA* ^*Lys*^	F	3725–3795	71	3755-3757CTT			0
*tRNA* ^*Asp*^	F	3801–3868	68	3833-3835GTC			5
*ATP8*	F	3869–4033	165		ATT	TAA	0
*ATP6*	F	4027–4704	678		ATG	TAA	-7
*COIII*	F	4704–5492	789		ATG	TAA	-1
*tRNA* ^*Gly*^	F	5499–5563	65	5529-5531TCC			6
*ND3*	F	5561–5917	357		ATA	TAA	-3
*tRNA* ^*Ala*^	F	5922–5987	66	5951-5953TGC			4
*tRNA* ^*Arg*^	F	5987–6050	64	6016-6018TCG			-1
*tRNA* ^*Asn*^	F	6077–6142	66	6107-6109GTT			26
*tRNA* ^*Ser(AGN)*^	F	6142–6211	70	6168-6170GCT			-1
*tRNA* ^*Glu*^	F	6211–6278	68	6241-6243TTC			-1
*tRNA* ^*Phe*^	R	6297–6363	67	6329-6331GAA			18
*ND5*	R	6364–8083	1720		ATT	T	0
*tRNA* ^*His*^	R	8099–8165	67	8133-8135GTG			15
*ND4*	R	8166–9504	1339		ATG	TA	0
*ND4L*	R	9498–9794	297		ATG	TAA	-7
*tRNA* ^*Thr*^	F	9797–9861	65	9827-9829TGT			2
*tRNA* ^*Pro*^	R	9862–9927	66	9895-9897TGG			0
*ND6*	F	9930–10454	525		ATT	TAA	2
*CytB*	F	10454–11590	1137		ATG	TAA	-1
*tRNA* ^*Ser(UCN)*^	F	11593–11659	67	11623-11625TGA			2
*ND1*	R	11676–12614	939		ATA	TAA	16
*tRNA* ^*Leu(CUN)*^	R	12625–12689	65	12658-12660TAG			10
*lrRNA*	R	12690–14019	1330				0
*tRNA* ^*Val*^	R	14020–14091	72	14056-14058TAC			0
*srRNA*	R	14092–14875	784				0
*Control region*		14876–16141	1266				0

^a^ Negative numbers indicate that adjacent genes overlap.

The gene order in the *D*. *antiqua* mitochondrial genome is the same as the gene order in *Dr*. *melanogaster*, which is the classical structure for Diptera [29]. The gene order of this mitochondrial genome shows the order is highly conserved in Diptera, and only in the Cecidomyiidae do we see the rearrangement in trnA and trnR forming trnR-trnA. Other known dipteran species all have the same gene order as *D*. *melanogaster*. Rearrangements of the mitochondrial genome are relatively rare as evolutionary events; therefore, this is an important tool to evaluate the phylogenetic relations between different species.

### Nucleotide Composition

The nucleotide composition of the mitochondrial genome of *D*. *antiqua* showed obvious bias towards A and T. The A+T content of the whole genome was 78.5% (A% = 39.6%, T% = 38.9%, G% = 8.9%, C% = 12.6%). The A+T content of isolated PCGs, tRNAs, rRNAs, control region and J-strand, N-strand were all above 70% (Table 2). The control region has the highest A+T content (93.7%). The skew statistics of the whole genome showed that the whole mitochondrial genome of *D*. *antiqua* is CG-skewed distinctly with almost equal A and T. The protein coding genes and rRNAs are TA-skewed and GC-skewed, tRNAs showed as AT-skewed and GC-skewed, the control region preferred to use T and C. Isolated genes on different strands showed different nucleotide bias (Table 2).

**Table 2:** Nucleotide composition of the *Delia antiqua* mitochondrial genome.

Feature	Proportion of nucleotides							
	%T	%C	%A	%G	%A+T	%G+C	AT Skew	GC Skew
Whole genome	38.9	12.6	39.6	8.9	78.5	21.5	0.01	-0.17
Protein-coding genes	43.9	11.5	32.5	12.1	76.4	23.6	-0.15	0.03
First codon position	33.4	9.7	43.0	13.9	76.4	23.6	0.13	0.18
Second codon position	43.2	14.5	28.0	14.3	71.2	28.8	-0.21	-0.01
Third codon position	46.0	10.2	35.8	8.0	81.8	18.2	-0.12	-0.12
Protein-coding genes-J	41.9	13.7	33.3	11.1	75.2	24.8	-0.11	-0.10
First codon position	39.9	8.9	41.0	10.2	80.9	19.1	0.01	0.07
Second codon position	40.0	17.5	27.3	15.2	67.3	32.7	-0.19	-0.07
Third codon position	45.3	14.6	32.6	7.5	77.9	22.1	-0.16	-0.32
Protein-coding genes-N	47.2	8.0	31.3	13.5	78.5	21.5	-0.20	0.26
First codon position	45.0	7.0	33.2	14.8	78.2	21.8	-0.15	0.36
Second codon position	48.2	9.1	30.0	12.7	78.2	21.8	-0.23	0.17
Third codon position	49.0	7.5	30.5	13.0	79.5	20.5	-0.23	0.27
tRNA genes	38.2	9.8	39.7	12.3	77.9	22.1	0.02	0.11
tRNA genes-J	38.2	11.2	39.0	11.6	77.2	22.8	0.01	0.02
tRNA genes-N	40.9	7.3	38.2	13.6	79.1	20.9	-0.03	0.30
rRNA genes	40.9	6.5	39.9	12.7	80.8	19.2	-0.01	0.32
Control region	47.9	4.1	45.8	2.2	93.7	6.3	-0.02	-0.30

**Fig 2:**
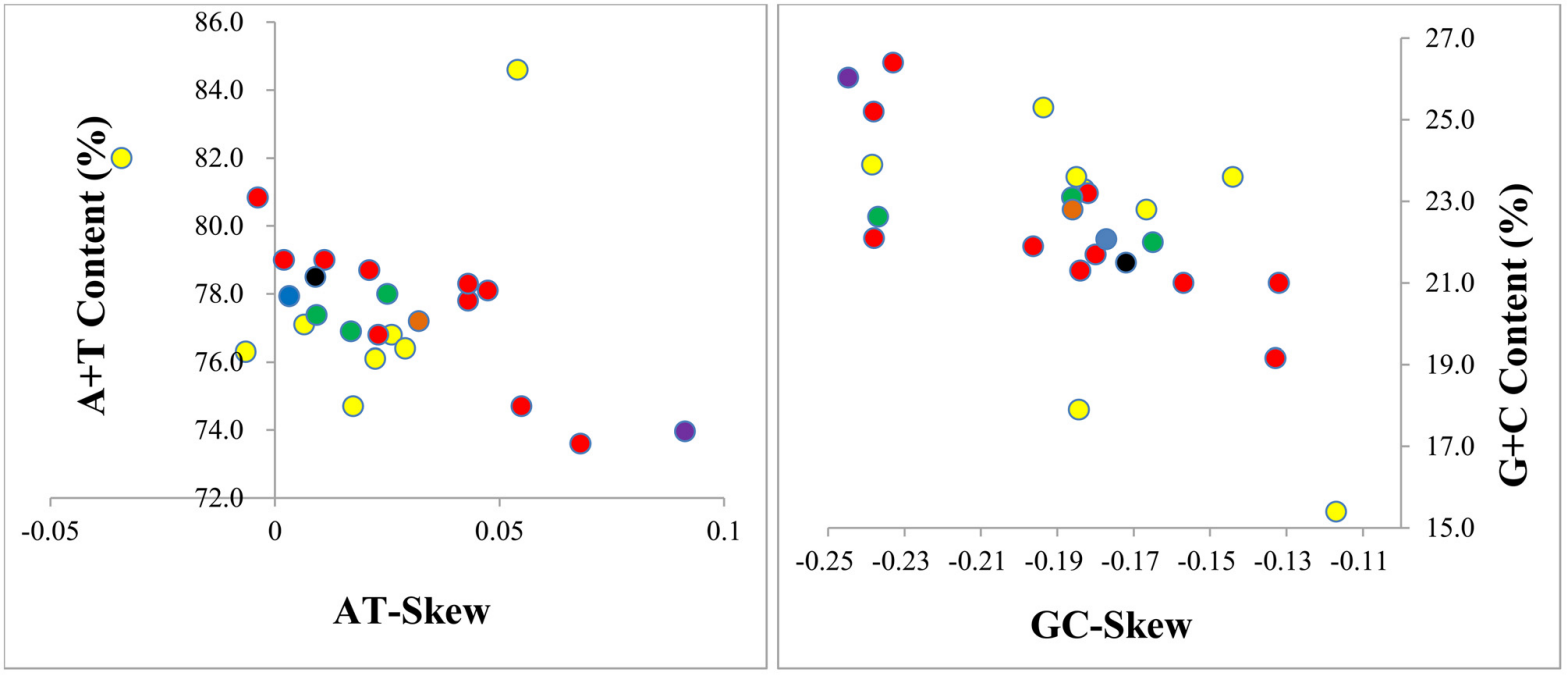
AT% vs AT-Skew and GC% vs GC-Skew in Diptera mitochondrial genomes. Measured in bp percentage (Y-axis) and level of nucleotide skew (X-axis). Values are calculated on full length mitochondrial genomes. Orange circle, Diptera; red circle, Muscomorpha; yellow circle, Tipulomorpha; Green circle, Culicomorpha; blue circle, Tabanomorpha; black circle, *Delia antiqua* (Anthomyiidae).

This strand bias in nucleotide composition is a universal phenomenon in metazoan mitochondrial genomes. The strand bias can be indicated by a comparative analysis of (A + T)% vs AT-skew and (G + C)% vs GC-skew. The mitochondrial genome analysis of all known families of Diptera is shown in Fig 2. The average AT-skew among the Diptera is 0.032, ranging from -0.034 in *Arachnocampa flava* to 0.131 in *Bactrocera minax*, whereas the *D*. *antiqua* mitochondrial genome shows a quite weak AT-skew (0.009) (Table 2). The average GC-skew among the Diptera is -0.186, ranging from -0.315 in *Bactrocera minax* to -0.110 in *Mayetiola destructo*, and the *D*. *antiqua* mitochondrial genome shows a little higher than the average value (-0.172) (Table 3). The AT-skew and GC-skew of most dipteran mitochondrial genomes shows a positive AT-skew and negative GC-skew for the J-strand. AT content and GC content consistently show that the dipteran mitochondrial genomes have higher percentages of A+T. The underlying mechanism of this bias has been generally related to asymmetric mutation and selection pressure during replication and transcription. In the process of DNA replication and transcription, one chain is a single chain longer than the other strand, the deamination rate of A and C is faster in single chain, and therefore, more deamination of A and C occurs, leading to this bias [30]. This nucleotide bias has significance for the study of replication, transcription and rearrangement of the mitochondrial genome.

**Table 3:** Base composition and strand bias across mitochondrial genomes of Dipteran families.

Family (number of species investigated)	A	T	AT-Skew	A+T	G	C	GC-Skew	G+C
Tipulomorpha								
Trichoceridae (2)	39.4	37.4	0.026	76.8	9.5	13.8	-0.183	23.3
Ptychopteridae (2)	39.3	37.1	0.029	76.4	9.6	14	-0.185	23.6
Anisopodidae (1)	37.9	38.4	-0.007	76.3	10.1	13.5	-0.144	23.6
Tanyderidae (1)	38.8	38.3	0.006	77.1	9.5	13.3	-0.167	22.8
Tipulidae (1)	38.0	36.7	0.017	74.7	10.2	15.1	-0.194	25.3
Cecidomyiidae (2)	44.6	40.0	0.054	84.6	6.8	8.6	-0.117	15.4
Pachyneuridae (1)	38.9	37.2	0.022	76.1	9.1	14.8	-0.238	23.9
Keroplatidae (1)	39.6	42.4	-0.034	82.0	7.3	10.6	-0.184	17.9
Muscomorpha								
Syrphidae (1)	40.3	40.6	-0.004	80.8	10.9	19.2	-0.133	19.2
Muscidae (2)	39.6	39.4	0.002	79.0	9.1	11.9	-0.132	21.0
Oestridae (2)	40.6	37.2	0.043	77.8	8.4	13.7	-0.238	22.1
Tachinidae (3)	40.2	38.5	0.021	78.7	8.7	12.6	-0.184	21.3
Agromyzidae (4)	40.9	37.5	0.043	78.3	8.9	12.8	-0.180	21.7
Tephritidae (12)	39.3	34.4	0.068	73.6	10.1	16.3	-0.233	26.4
Calliphoridae (13)	39.3	37.5	0.023	76.8	9.5	13.7	-0.182	23.2
Drosophilidae (18)	40.0	39.0	0.011	79.0	8.8	12.2	-0.157	21.0
Sarcophagidae (1)	39.4	35.3	0.055	74.7	9.6	15.6	-0.238	25.2
Fergusoninidae (1)	40.9	37.2	0.047	78.1	8.8	13.1	-0.196	21.9
Anthomyiidae (1)	39.6	38.9	0.009	78.5	8.9	12.6	-0.172	21.5
Culicomorpha								
Culicidae (16)	40.0	38	0.025	78.0	9.2	12.8	-0.165	22.0
Ceratopogonidae (1)	39.0	38.3	0.009	77.4	8.6	14.0	-0.237	22.6
Chironomidae (1)	39.1	37.8	0.017	76.9	9.4	13.7	-0.186	23.1
Tabanomorpha								
Tabanidae (1)	39.1	38.8	0.003	77.9	9.1	13.0	-0.177	22.1
Asilomorpha								
Nemestrinidae (1)	40.4	33.6	0.091	74.0	9.8	16.2	-0.245	26.0
Avg.	39.8	37.4	0.032	77.2	9.3	13.6	-0.186	22.8

### Protein-coding Genes

Most of the protein-coding genes use ATN as start codon (four use ATT, six use ATG, and two use ATA). The only exception is the *COI* gene, which begins with the special quadruplet start codon of ATCA (Table 1). Only *COII*, *ND5* and *ND4* genes had incomplete termination codons of T and TA, all others use the complete termination codons TAA (*ND2*, *COI*, *ATP8*, *ATP6*, *COIII*, *ND3*, *ND6*, *ND4L*, *ND1*, *CytB*) (Table 1).

The nucleotide bias is also reflected in the protein-coding genes. The base composition of each codon position for the 13 protein-coding genes shows that they all have a high A+T percentage. The third codon position (81.8%) was distinctly higher than the other two codon positions (76.4% and 71.2%). The A+T content of the protein-coding genes on different strands also show a high percentage (Table 2). Different codon positions of protein-coding genes show different skew statistics. The first codon position prefers to use A and G, and the others were TA-skewed and CG-skewed. The genes on the J-strand and in its second and third codon position all showed TA-skew and CG-skew; the first codon position was AT-skewed and GC-skewed; the genes on the N-strand all had a higher frequency of T and G (Table 2).

The bias of amino acids was found in the protein-coding genes. The protein-coding genes and genes on different strands all had an unbalanced percentage of amino acids. They all had a high percentage of Leu, and the least percentage of Cys (Table 4). The relative synonymous codon usage also showed significant biases. The most frequently used codons were UUA, CGA, GGA, GCU, UCA and GUA, with the codons CUC, CUG, CCG, ACG, GGC and GCG most rarely used (Table 5).

**Table 4:** The percentage of amino acid for *Delia antiqua* mitochondrial genome.

Feature	Percentage (%)		
	Protein-coding genes	Protein-coding genes-J	Protein-coding genes-N
Ala	4.43	4.53	4.27
Cys	0.99	0.52	1.75
Asp	1.80	1.96	1.54
Glu	2.04	1.96	2.17
Phe	9.21	8.94	9.66
Gly	5.80	5.71	5.95
His	1.91	2.57	0.84
Ile	9.70	10.55	8.33
Lys	2.42	2.27	2.66
Leu	16.20	14.82	18.40
Met	6.31	5.67	7.35
Asn	5.35	5.67	4.83
Pro	3.65	4.45	2.38
Gln	2.04	2.22	1.75
Arg	1.50	1.61	1.33
Ser	9.13	8.46	10.22
Thr	5.10	6.32	3.15
Val	5.26	4.97	5.74
Trp	2.60	2.96	2.10
Tyr	4.51	3.84	5.60

**Table 5:** Relative synonymous codon usage (RSCU) in the *Delia antiqua* mitochondrial genome.

Codon	RSCU	Codon	RSCU	Codon	RSCU	Codon	RSCU
UUU(F)	1.63	UCU(S)	1.73	UAU(Y)	1.63	UGU(C)	1.38
UUC(F)	0.37	UCC(S)	0.28	UAC(Y)	0.37	UGC(C)	0.62
UUA(L)	4.06	UCA(S)	2.13	UAA(*)	1.33	UGA(W)	1.37
UUG(L)	0.57	UCG(S)	0.24	UAG(*)	0.67	UGG(W)	0.63
CUU(L)	0.65	CCU(P)	1.71	CAU(H)	1.59	CGU(R)	0.44
CUC(L)	0.17	CCC(P)	0.71	CAC(H)	0.41	CGC(R)	0.18
CUA(L)	0.41	CCA(P)	1.42	CAA(Q)	1.75	CGA(R)	3.02
CUG(L)	0.13	CCG(P)	0.17	CAG(Q)	0.25	CGG(R)	0.36
AUU(I)	1.78	ACU(T)	1.58	AAU(N)	1.59	AGU(S)	1.16
AUC(I)	0.22	ACC(T)	0.59	AAC(N)	0.41	AGC(S)	0.59
AUA(M)	1.57	ACA(T)	1.72	AAA(K)	1.53	AGA(S)	1.14
AUG(M)	0.43	ACG(T)	0.11	AAG(K)	0.47	AGG(S)	0.72
GUU(V)	1.58	GCU(A)	2.25	GAU(D)	1.81	GGU(G)	1.06
GUC(V)	0.2	GCC(A)	0.38	GAC(D)	0.19	GGC(G)	0.14
GUA(V)	2.03	GCA(A)	1.29	GAA(E)	1.7	GGA(G)	2.36
GUG(V)	0.2	GCG(A)	0.08	GAG(E)	0.3	GGG(G)	0.44

In the dipteran mitochondrial genomes, *COI* initiation codons are variable and include TCG, CCG, ATCA and ATTTAA [31–33]. It is a common phenomenon to use an incomplete codon as a termination codon. They will be supplemented by processing after transcription [34]. This bias is also reflected in the codon usage and amino acid composition. The protein-coding genes of the *D*. *antiqua* mitochondrial genome prefer to use codon UUA (Leu) and Leucine. This is expected because there are many transmembrane proteins in the mitochondrial genome and Leucine happens to be a kind of hydrophobic amino acid.

### Transfer RNAs

**Fig 3:**
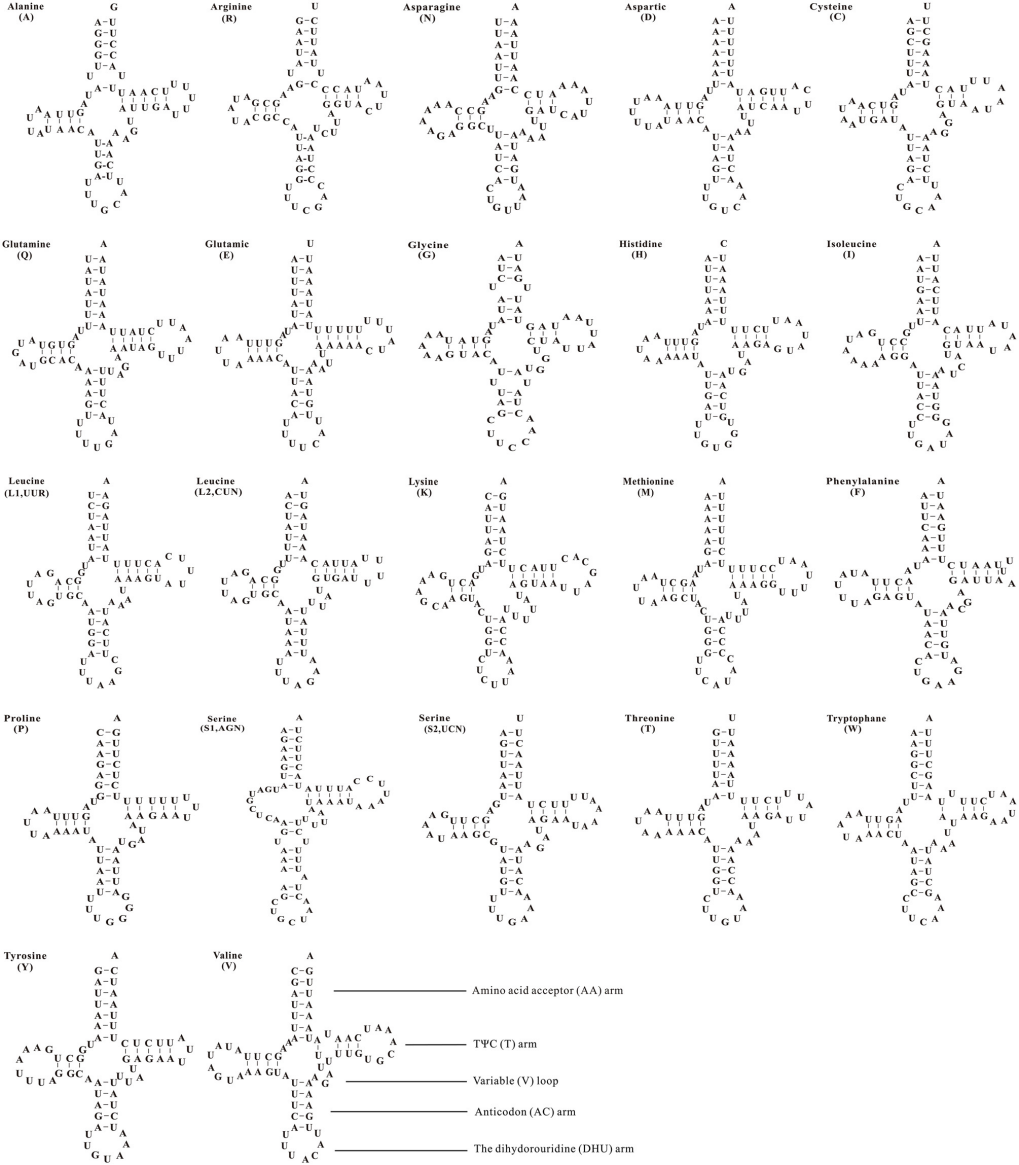
Inferred secondary structure of tRNAs in the *Delia antiqua* mitochondrial genome. The tRNAs are labeled with their corresponding amino acids.

Twenty-two complete tRNAs were found in the *D*. *antiqua* mitochondrial genome, and 20 of them were identified by tRNAscane-SE [35]. Only the *tRNA* ^*Arg*^ and *tRNA* ^*Ser(AGN)*^ could not be detected by software, and they were determined through comparison with published dipteran mitochondrial genomes. All tRNAs were folded into the typical clover-leaf structure except for *tRNA* ^*Ser(AGN)*^ (Fig 3). All tRNAs ranged from 63 to 72 bp in length. The typical clover-leaf structure contains an amino acid arm (7 bp), TΨC arm (3–5 bp), DHU arm (3–4 bp), anticodon arm (4–5 bp) and a variable extra arm. *tRNA* ^*Ser(AGN)*^ had a special clover-leaf structure without a DHU arm.

Based on the secondary structure of the tRNAs in the *D*. *antiqua* mitochondrial genome, there were 7 unmatched base pairs. All of them were U-U unmatched base pairs which were present in the amino acid arms, TΨC arm and anticodon arm.

### Ribosomal RNAs

The boundaries of rRNA genes were identified by sequence alignment with published dipteran sequences. There were two rRNA genes in the *D*. *antiqua* mitochondrial genome, *16S rRNA* and *12S rRNA*. The locations of the *16S rRNA* and *12S rRNA* genes were between *tRNA* ^*Leu(CUN)*^ and *tRNA* ^*Val*^ and between *tRNA* ^*Val*^ and the A+T-rich region, respectively. The *16S rRNA* gene is 1,330 bp long, and the *12 S rRNA* is 784 bp long. Their A+T content was 82.26% and 78.32%, respectively. The location of the two rRNAs is same as in other dipteran mitochondrial genomes and they are very conservative.

### The Control Region

The control region of the *Delia antiqua* mitochondrial genome is located between *12S rRNA* and *tRNA* ^*Ile*^ and is 1266 bp in length with the highest A+T content 93.7% of the whole genome. Three conserved structural elements have been identified in the control region of the *D*. *antiqua* mitochondrial genome. We found two poly-T stretches, one (TA)n stretch with 98 repeats and several G(A)nT structures by using the Tandem Repeats Finder [36]. One of the two poly-T stretches was found near the *tRNA* ^*Ile*^ gene in the minority strand with 37 bp; the other was located close to the *12S rRNA* which is in the majority strand and 27 bp in length. The (TA)n stretch was located in J-strand and the G(A)nT structures were on N-strand.

Five conserved special structures in the control region have been identified in insects: a poly-T stretch, a [TA(A)]n-like stretch, a highly conserved stem-and-loop structure, a G(A)nT structure, and a G+A-rich stretch [5]. But the five conserved structures are not all found in every insect [37–38]. In the control region of *D*. *antiqua*, three of these structures were found and they may be involved in the control of transcription or replication [39].

### Phylogenetic Relationships

**Fig 4:**
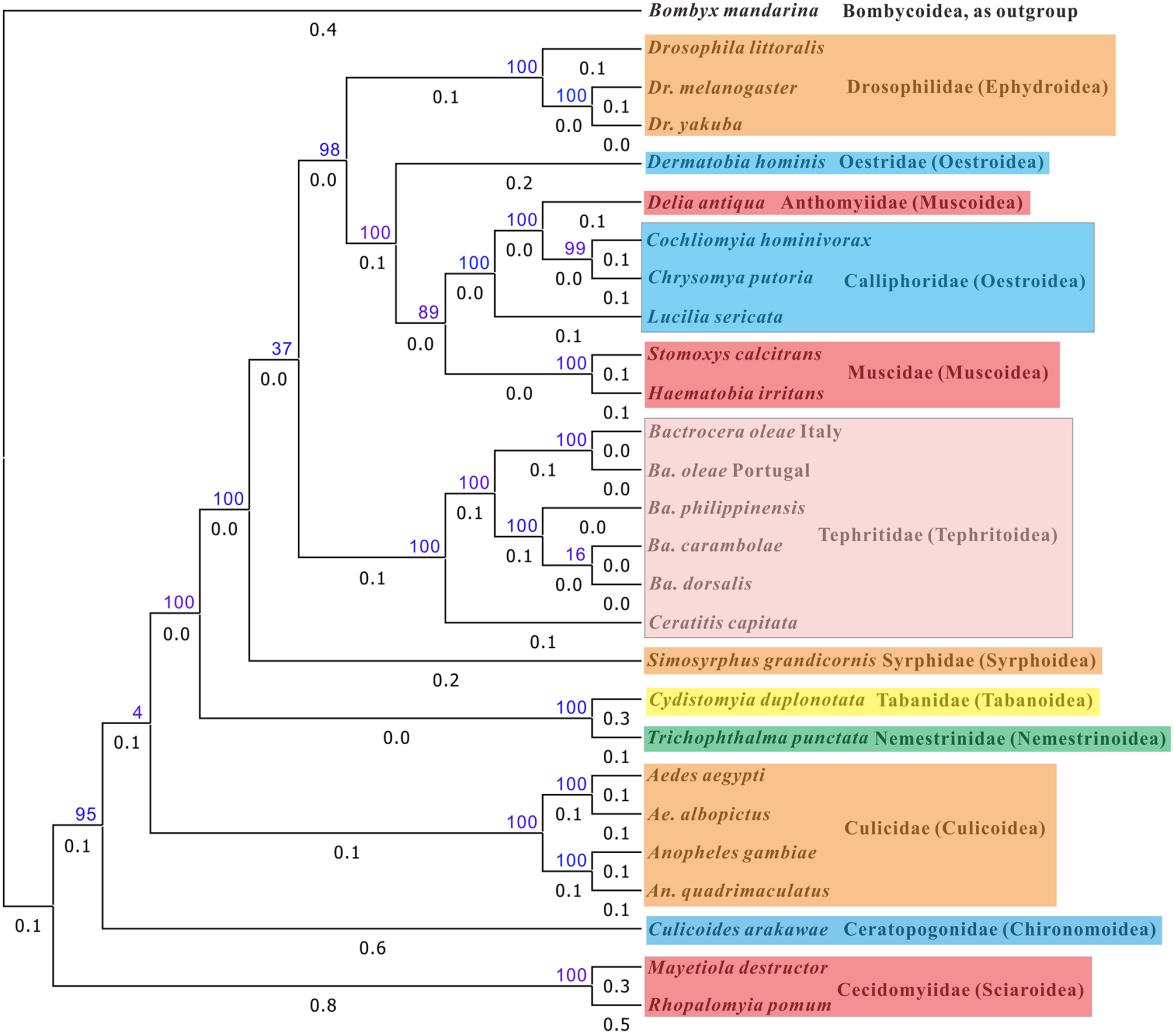
Phylogenetic tree of Diptera mitochondrial genome sequences inferred by Maximum Likelihood analysis. Numbers above branches and with blue color indicate likelihood bootstrap values, and the numbers below branches indicate the branch length. The names of family and superfamily (in bracket) are just after the species names.

**Fig 5:**
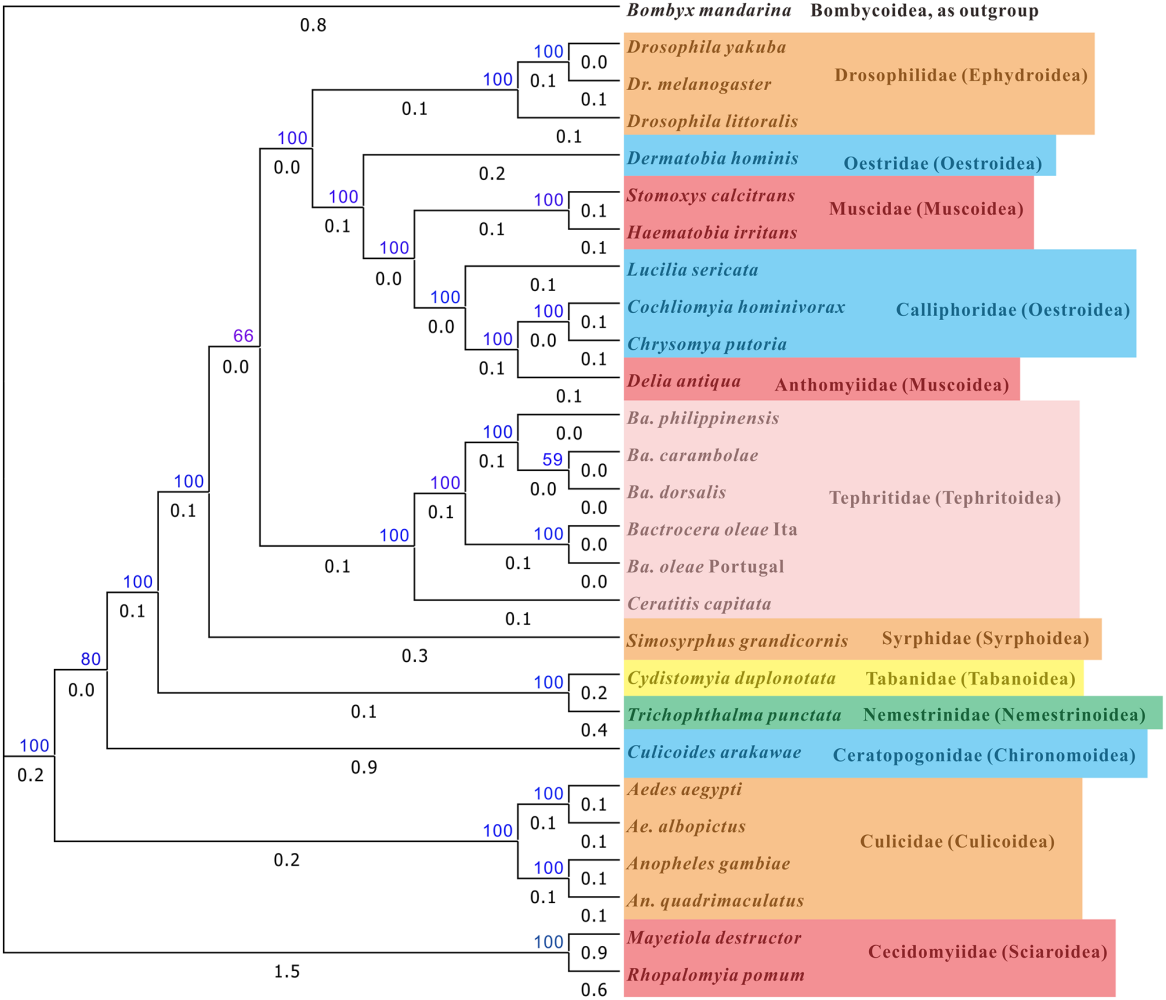
Phylogenetic tree of Diptera mitochondrial genome sequences inferred by Bayesian analysis. Numbers above branches and with blue color indicate likelihood bootstrap values, and the numbers below branches indicate the branch length. The names of family and superfamily (in bracket) are just after the species names.

We performed phylogenetic analysis using the nucleotide sequences of 13 protein-coding genes of 25 species of complete dipteran mitochondrial genome sequences and the *D*. *antiqua* mitochondrial genome using *Bombyx mandarina* as outgroup. The topological strctures of the 2 phylogenetic trees constructed separately by ML and BI analyses are very similar, with only 1 exception of the location of *Culicoides arakawae* in the family Ceratopogonidae of the superfamily Chironomoidea (Figs 4 and 5). On the ML tree the species is located at the base of the Culicidae (Culicoidea) clade (Fig 4), whereas on the BI tree it is linked up the Culicidae clade (Fig 5). All but 3 clades are strongly supported with >80 bootstrap values. However, the clade of Ephydroidea + Oestroidea + Muscoidea + Tephritoidea and the clade of *Ba*. *carambolae* + *Ba*. *dorsalis* in Tephritidae are with bootstrap values equal or less 66 on both trees. On the Ml tree the clade up *Culicoides arakawae* has only a bootstrap value of 4, and on the BI tree the clade below *Culicoides arakawae* has a bootstrap value of 88, which indidates that the location of *Culicoides arakawae* is pending.

More importanly, *D*. *antiqua* of Anthomyiidae (Muscoidea) is nested inside Calliphoridae (Oestroidea) clade, and Muscidae (Muscoidea) is linked inside the Oestroidea. Kutty et al. (2008) constrcted the phylogenetic trees of Muscoidea, Hippoboscoidea and Oestroidea using 4 mitochondrial genes *12S*, *16S*, *COI*, and *Cytb*, and 4 nuclear genes *18S*, *28S*, *Ef1a* and CAD [15]. The results showed that the Muscoidea is paraphyletic with a monophyletic Oestroidea nested within the Muscoidea as sister group to Anthomyiidae + Scathophagidae, the Anthomyiidae is possibly paraphyletic, and the Calliphoridae is paraphyletic. Marinho et al. (2012) inferred the phylogenetic relationship of families in the Oestroidea using ITS2, 28S, COI and 16S regions, and suggest that Calliphoridae is paraphyletic [16]. Nelson et al. (2012) constructed the phylogenetic tree of 13 Calliphoridae species of whole mtgenome sequences using 13 protein-coding genes and 2 ribosomal RNA genes, and suggest that Calliphoridae is polyphyletic [17]. The present study suggest a closer relationship of Anthomyiidae with Calliphoridae, but more whole mtgenome sequences are necessary to elucidate its paraphyly and phylogenetic diversity inside the family. The study also suggest that Calliphoridae is a paraphyly, and further study might elucidate the tranditional taxonomy of Anthomyiidae and Calliphoridae. The study suggest that both Oestroidea and Muscoidea are polyphyletic, which are partially supported by Kutty et al. (2008) and Nelson et al. (2012) [15, 17].

## Conclusions

This is the first report of complete mitochondrial genome of the family Anthomyiidae. Comparative analysis showed that the gene size, gene order, base content, and base composition are comparatively conserved as with other dipteran mitochondrial genomes. All of the 13 protein-coding genes use ATN as the initiation codon except for the *COI* gene, which starts with ATCA. Most tRNAs have the typical clover-leaf structure, except *tRNA* ^*Ser(AGN)*^, which does not contain the dihydrouridine (DHU) arm. The location and structure of the two rRNAs are conservative and comparable with Dipteran and other insects. The control region between *12S rRNA* and *tRNA* ^*Ile*^ has the highest A+T content 93.7% in the *D*. *antiqua* mitochondrial genome. There were three kinds of special structures found in the control region, poly-T stretches, a (TA)_n_ stretch and G(A)_n_T structures, which are considered as important elements related to replication and transcription.

Both maximum likelihood and Bayesian inference analyses using nucleotide sequences of 13 protein-coding genes highly suggest a closer relationship of *Delia antiqua* in Anthomyiidae has a closer with Calliphoridae, Calliphoridae is a paraphyly, and both Oestroidea and Muscoidea are polyphyletic. The whole mtgenome sequences have also been demonstrated as an effective method for resolving phylogenetic relationships [17, 40, 41].

## Supporting Information

S1 Table The dipteran species used for this phylogenetic study with Bombyx mandarina of Lepidoptera as outgroup. (DOC)
