# Supplementary material for: The Complete Mitochondrial Genome of Delia antiqua and Its Implications in Dipteran Phylogenetics
Source: PLoS One. 2015 Oct 1;10(10):e0139736. doi: 10.1371/journal.pone.0139736 (PMC4591329; doi:10.1371/journal.pone.0139736)
Supplement: S1 Table — (DOC) [file pone.0139736.s001.doc]

**Supporting Information Table 1.** The dipteran species used for this phylogenetic study with *Bombyx mandarina* of Lepidoptera as outgroup.

| Family (Superfamily) | Species | Access No. |
| --- | --- | --- |
| Drosophilidae (Ephydroidea) | *Drosophila littoralis* | NC_001709 |
|  | *Dr. melanogaster* | NC_011596 |
|  | *Dr. yakuba* | NC_001322 |
| Oestridae (Oestroidea) | *Dermatobia hominis* | NC_006378 |
| Anthomyiidae (Muscoidea) | *Delia antiqua* | KT026595 |
| Calliphoridae (Oestroidea) | *Cochliomyia hominivorax* | NC_002660 |
|  | *Chrysomya putoria* | NC_009733 |
|  | *Lucilia sericata* | NC_002697 |
| Muscidae (Muscoidea) | *Stomoxys calcitrans* | DQ533708 |
|  | *Haematobia irritans* | NC_007102 |
| Tephritidae (Tephritoidea) | *Ceratitis capitata* | NC_009772 |
|  | *Bactrocera oleae* Italy | NC_008748 |
|  | *Ba. oleae* Portugal | NC_009771 |
|  | *Ba. philippinensis* | AY_210703 |
|  | *Ba. carambolae* | NC_005333 |
|  | *Ba. dorsalis* | NC_000857 |
| Syrphidae (Syrphoidea) | *Simosyrphus grandicornis* | NC_008754 |
| Tabanidae (Tabanoidea) | *Cydistomyia duplonotata* | NC_008756 |
| Nemestrinidae (Nemestrinoidea) | *Trichophthalma punctata* | NC_008755 |
| Culicidae (Culicoidea) | *Aedes aegypti* | NC_010241 |
|  | *Ae. albopictus* | NC_006817 |
|  | *Anopheles gambiae* | NC_002084 |
|  | *An. quadrimaculatus* | NC_000875 |
| Ceratopogonidae (Chironomoidea) | *Culicoides arakawae* | NC_009809 |
| Cecidomyiidae (Sciaroidea) | *Mayetiola destructor* | GQ387648 |
|  | *Rhopalomyia pomum* | GQ387649 |
| [Bombycidae](http://en.wikipedia.org/wiki/Bombycidae) (Bombycoidea, as outgroup) | *Bombyx mandarina* | NC_003395 |
